# Supplementary material for: Enabling low-drift flexible perovskite photodetectors by electrical modulation for wearable health monitoring and weak light imaging
Source: Nat Commun. 2023 Aug 16;14:4961. doi: 10.1038/s41467-023-40711-1 (PMC10432415; doi:10.1038/s41467-023-40711-1)
Supplement: Supplementary file 1 — Supplementary Information [file 41467_2023_40711_MOESM1_ESM.pdf]

Supplementary Information

Enabling low-drift flexible perovskite photodetectors by electrical modulation for wearable health monitoring and weak light imaging

Yingjie Tang[1,2†], Peng Jin[3†], Yan Wang[1,2], Dingwei Li[1,2], Yitong Chen[2,4], Peng Ran[3], Wei Fan[2,4], Kun Liang[1,2], Huihui Ren[2,4], Xuehui Xu[3], Rui Wang[2,5], Yang (Michael) Yang[3]*, Bowen Zhu[2,5]*

†Those authors contributed equally to this work.

1. College of Information Science and Electronic Engineering, Zhejiang University, Hangzhou 310027, China.
2. Key Laboratory of 3D Micro/Nano Fabrication and Characterization of Zhejiang Province, School of Engineering, Westlake University, Hangzhou 310024, China.
3. State Key Laboratory of Modern Optical Instrumentation, College of Optical Science and Engineering, Zhejiang University, Hangzhou 310007, Zhejiang, China.
4. School of Materials Science and Engineering, Zhejiang University, Hangzhou 310027, China.
5. Institute of Advanced Technology, Westlake Institute for Advanced Study, Hangzhou 310024, China.

*Corresponding authors.
Email: zhubowen@westlake.edu.cn (B.Z.); yangyang15@zju.edu.cn (Y.Y.)

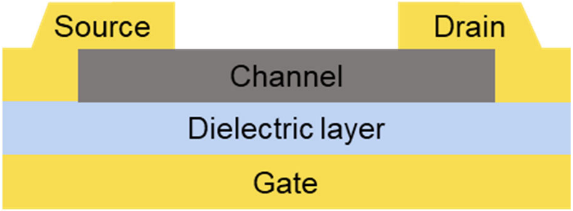

Supplementary Figure 1 | Schematic of a bottom gate top contact (BGTC) transistor.

Compared with the field-effect transistor (FET) configuration, our proposed structure has no dielectric layer, and the control electrode is in direct contact with the semiconductor material. The control electrode in our structure can directly participate in the current transport process between the source and drain electrodes, so it has a stronger control effect on the dark current, and the value of the dark current can be reduced to zero or even a negative value. Therefore, our structure can achieve lower dark current values compared to a transistor configuration.

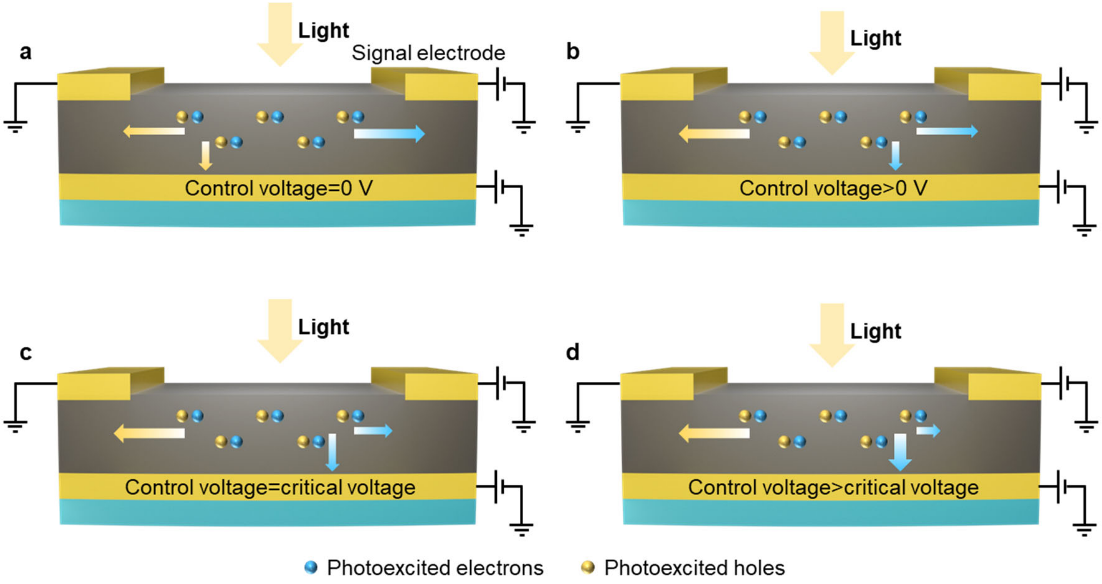

Supplementary Figure 2 | The photoexcited carriers (electrons and holes) transport process under different applied control voltage. a, When the control voltage is set as 0 V, the photoexcited electrons are transported to the signal electrode and the photoexcited holes are transported to the ground and control electrode. **b-d**, When the control voltage increases (> 0 V), some photoexcited electrons will be attracted by the control electrode. The transport of photogenerated electrons depends on the competition between the control voltage and the working voltage. Overall, the SNR of the signal electrode can be significantly improved when the control voltage is set as CV (**c**).

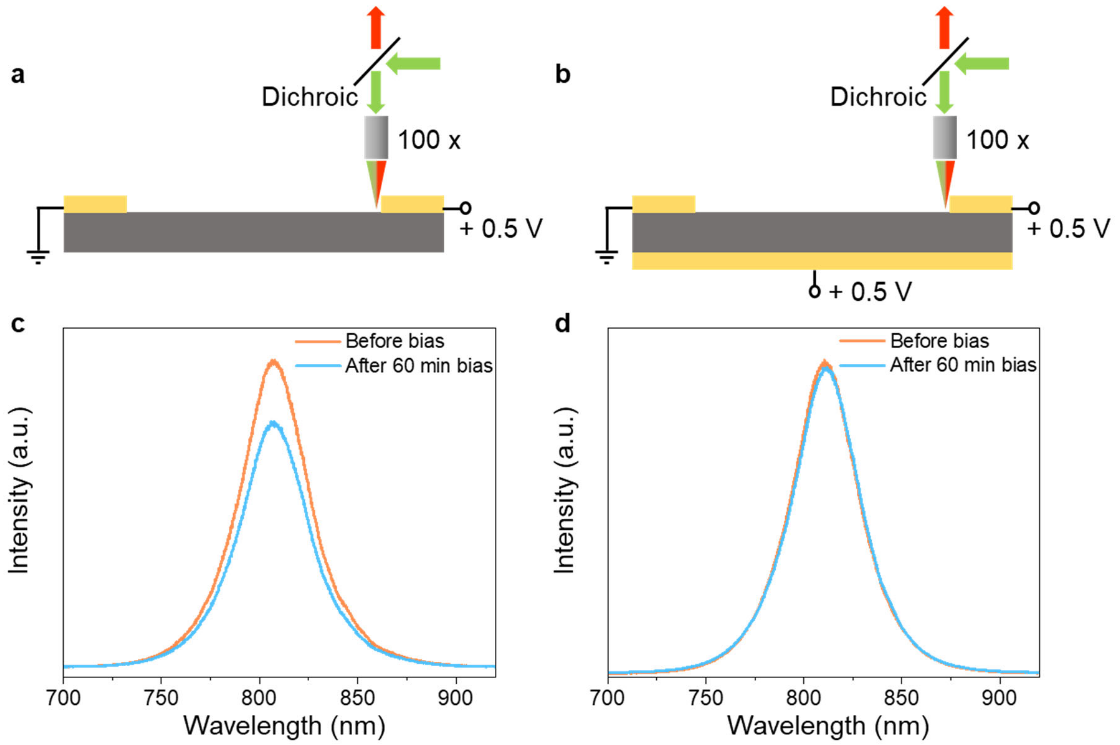

Supplementary Figure 3 | Measuring the photoluminescence intensity around the signal electrode. a,**b**, Schematic of the *in situ* PL intensity measurement around signal electrodes of conventional photoconductive-type (**a**) and electrical field modulated (**b**) FPDs. **c**,**d**, Measured PL intensity spectra near the signal electrode edges of photoconductive-type (**c**) and electrical field modulated (**d**) FPDs. a.u., arbitrary units.

After 0.5 V bias for 60 min, the PL intensity of conventional photoconductive-type FPD decreased significantly, while the PL intensity of the electrical field modulated FPD was almost unchanged, which proves that our method has a significant inhibitory effect on ion migration.

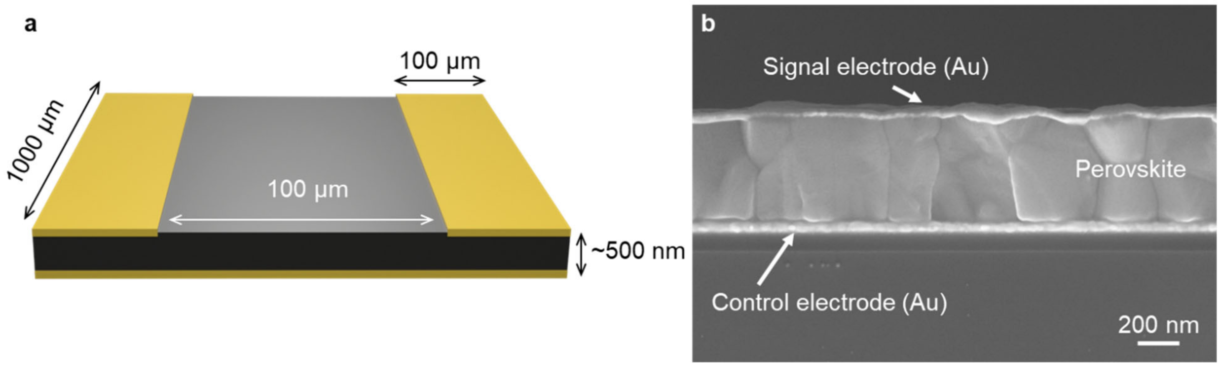

Supplementary Figure 4 | Configuration of the MHPs-based FPD with CE. a, A schematic showing the structure of electrical-field-modulated FPD. **b**, Cross-sectional SEM image of the perovskite fabricated on top of the control electrode. There is intimate contact between the perovskite and the Au control electrode without observable voids, implying good quality of the perovskite film.

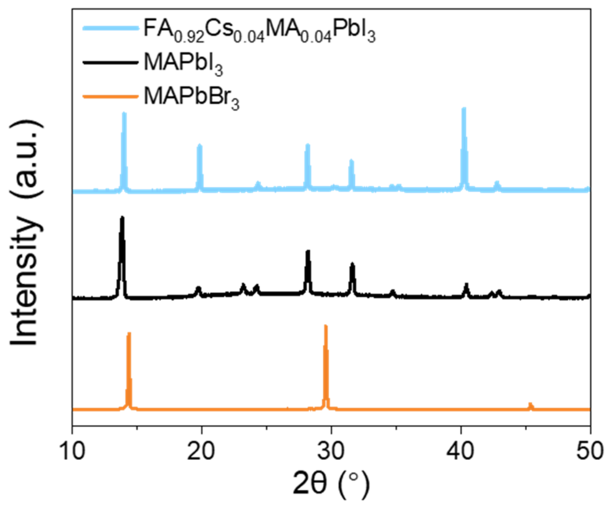

Supplementary Figure 5 | The XRD spectra of $FA_{0.92}Cs_{0.04}MA_{0.04}PbI_3$, $MAPbI_3$, and $MAPbBr_3$ films.

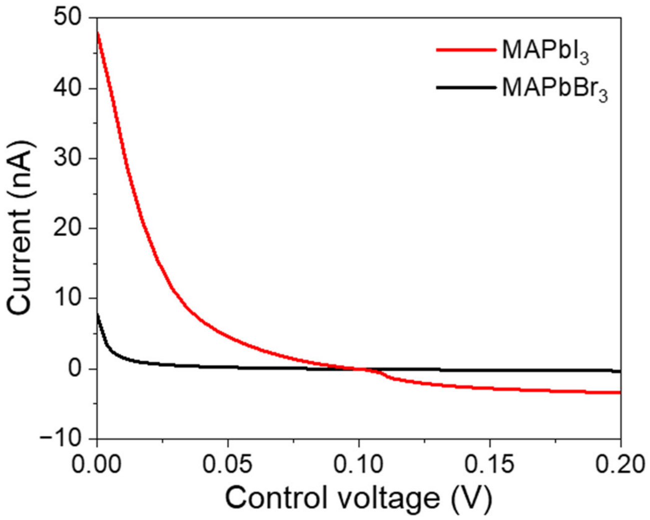

Supplementary Figure 6 | Dark current collected by the signal electrode of the $MAPbI_3$ and $MAPbBr_3$ based FPD (thickness=500 nm) varies with the control voltage when the signal electrode is set as 0.1 V (Critical voltage=0.1 V).

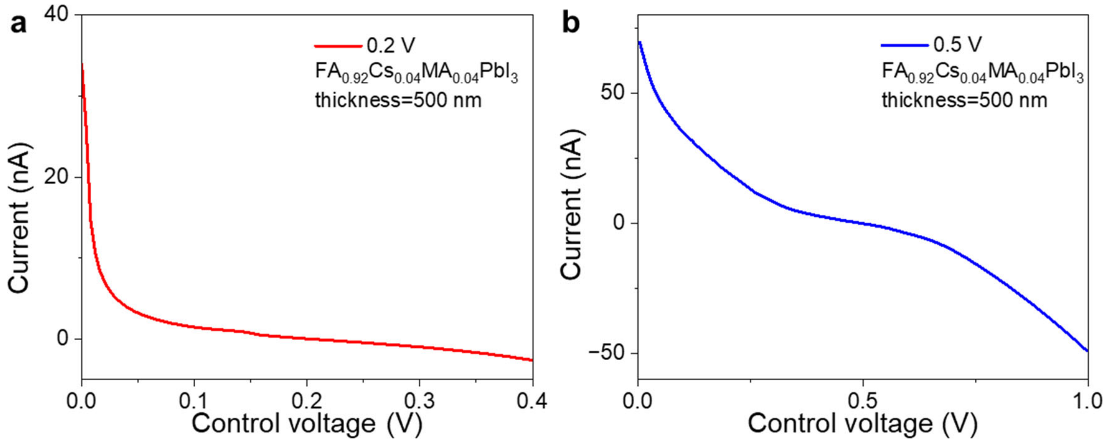

Supplementary Figure 7 | The dark current variation of FPD along with control voltage under specific driving voltage values (0.2, and 0.5 V). The corresponding critical voltage was 0.2 V (**a**) and 0.5 V (**b**), respectively.

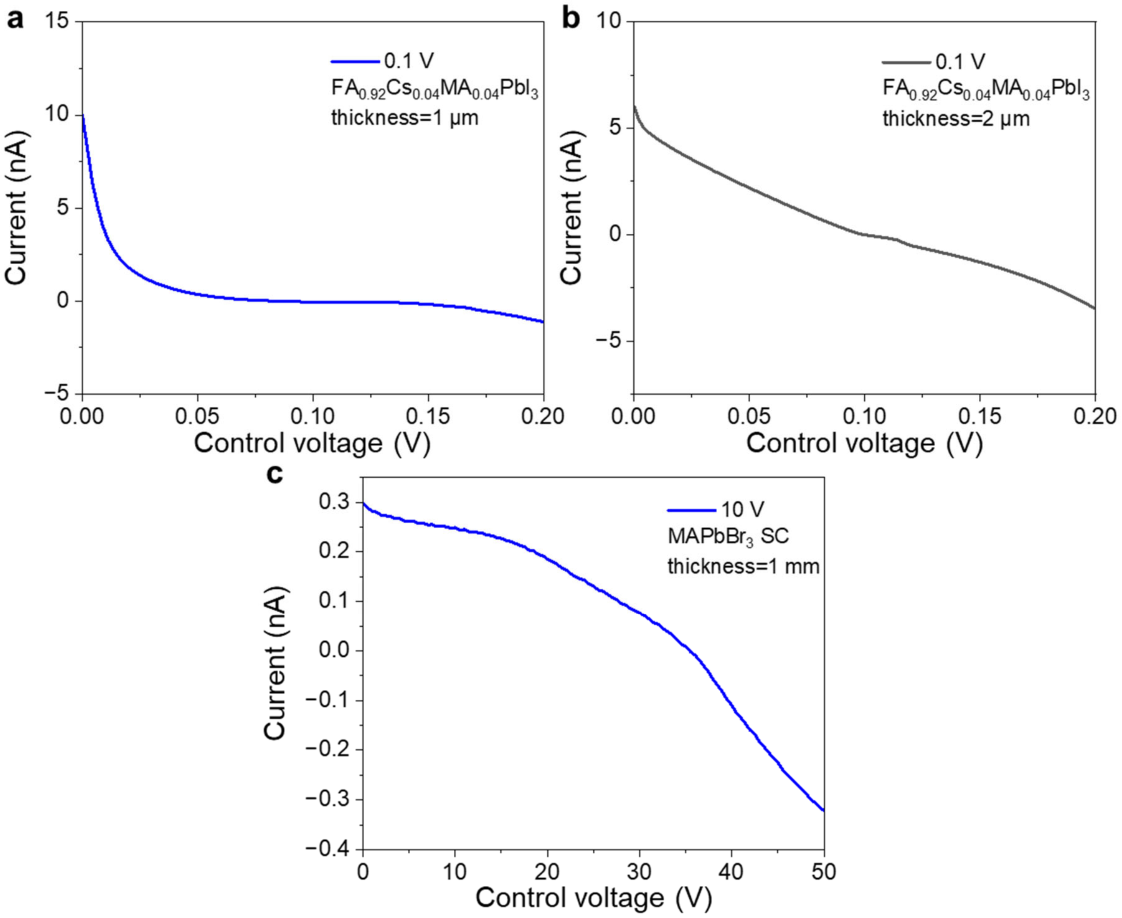

Supplementary Figure 8 | Current-voltage curves in terms of dark current collected by the signal electrode and control voltage. a-c, The FPDs with 1 μm $FA_{0.92}Cs_{0.04}MA_{0.04}PbI_3$ film (**a**) 2 μm $FA_{0.92}Cs_{0.04}MA_{0.04}PbI_3$ film (**b**) and 1 mm $MAPbBr_3$ SC (single crystal) (**c**). When the thickness of perovskite reaches 1mm, the critical voltage (35 V) is greater than the working voltage (10 V).

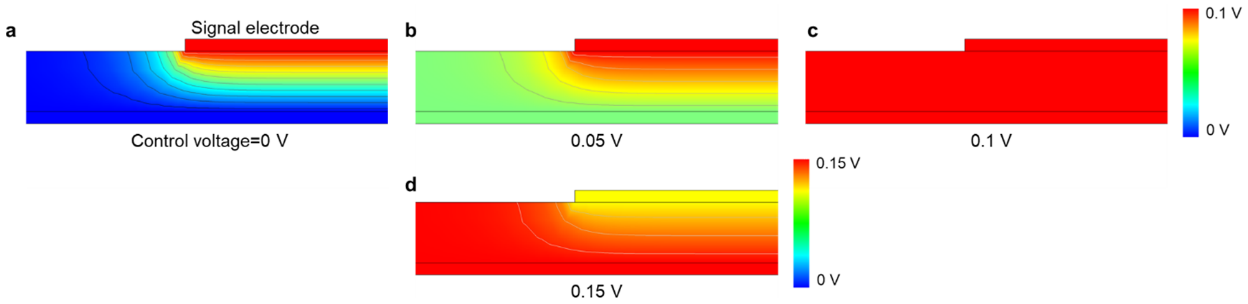

Supplementary Figure 9 | Electric field distributions in perovskite film (thickness=500 nm) under different control voltages (0, 0.05, 0.1 and 0.15 V) when the working voltage is 0.1 V.

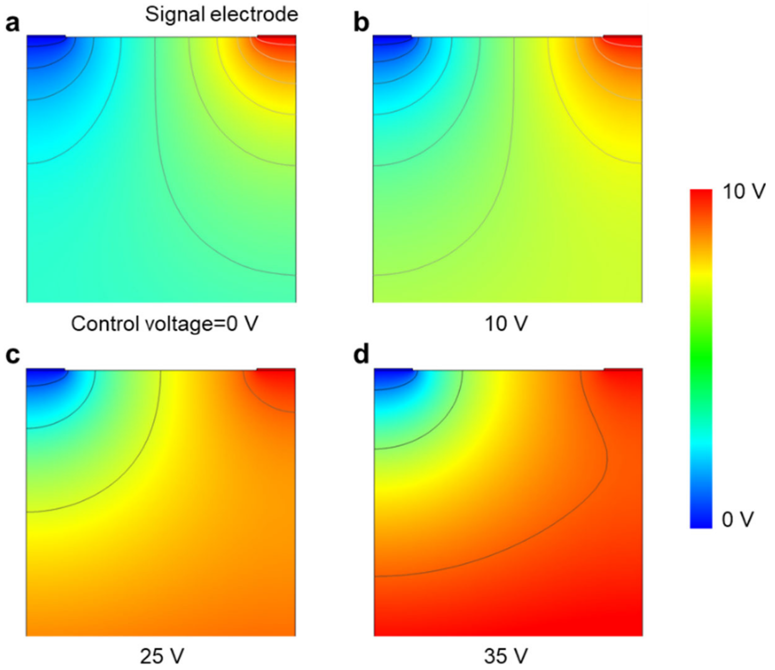

Supplementary Figure 10 | Electric field distributions in perovskite film (thickness=1 mm) under different control voltage (0, 10, 25 and 35 V) when the working voltage is 10 V.

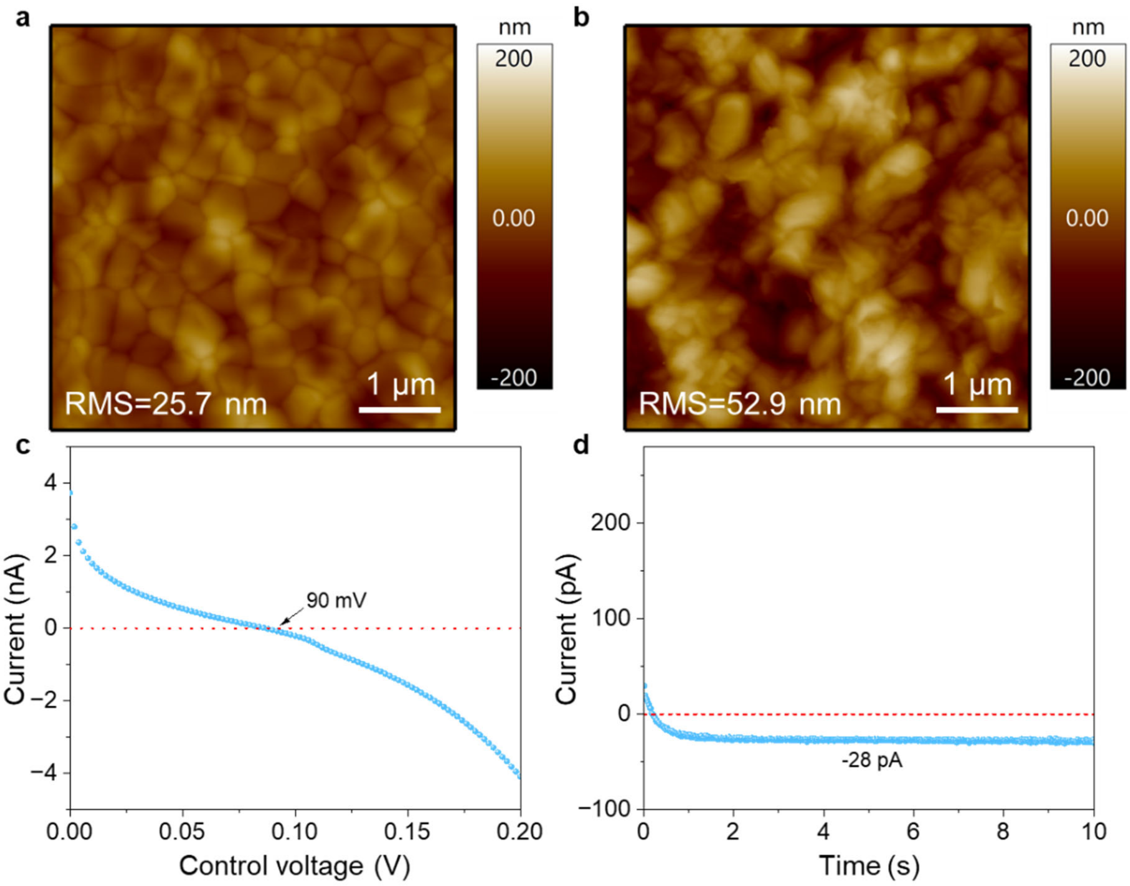

Supplementary Figure 11 | Effect of perovskite film quality on the voltage selection of control electrode. a,b, Surface morphologies of perovskite films fabricated with different antisolvent addition times. The root mean square (RMS) surface roughness of perovskite film increases significantly from 25.7 nm (**a**) to 52.9 nm (**b**), when the antisolvent CB addition time changes from 5 to 25 s countdown. **c,d**, Electrical properties of perovskite films with higher surface roughness. (**c**) Dark current varies with the control voltage when the working voltage is set to 0.1 V (critical voltage=90 mV). (**d**) *I-t* curve of the signal electrode under 0.1 V control voltage (dark current=-28 pA).

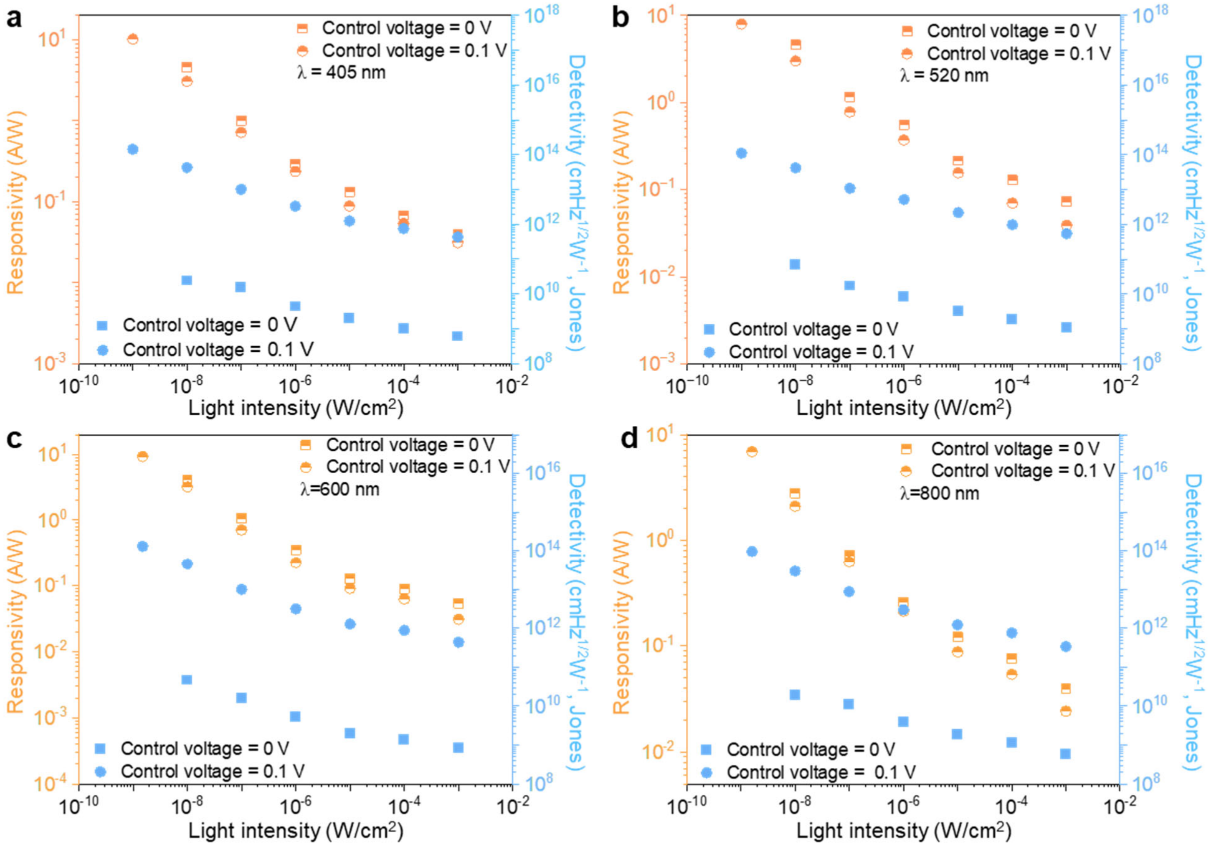

Supplementary Figure 12 | Dependence of responsivity and detectivity on the light intensity at wavelengths of 405 (a), 520 (b), 600 (c), and 800 nm (d).

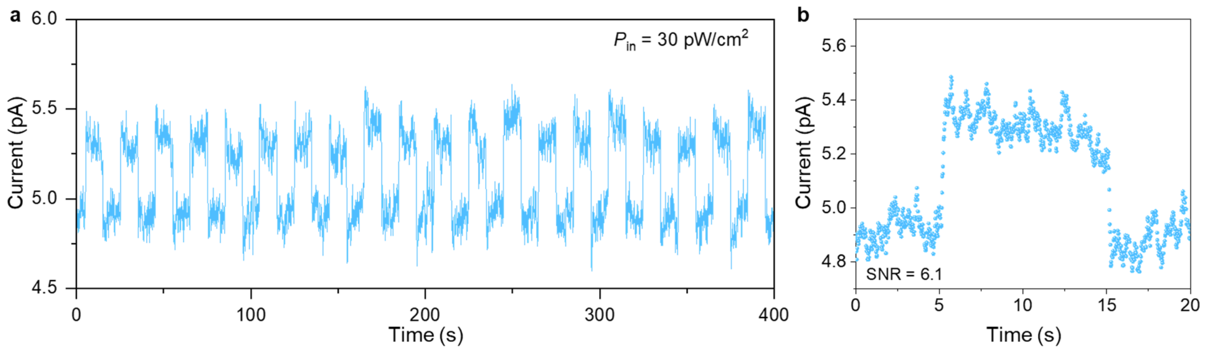

Supplementary Figure 13 | Time-resolved photocurrent response of FPD under weak light illumination (520 nm, 30 pW/cm^2) when the control voltage was 0.1 V. a, Twenty representative switching cycles of the FPD show good stability. **b**, Enlarged response pulse waveform in one period.

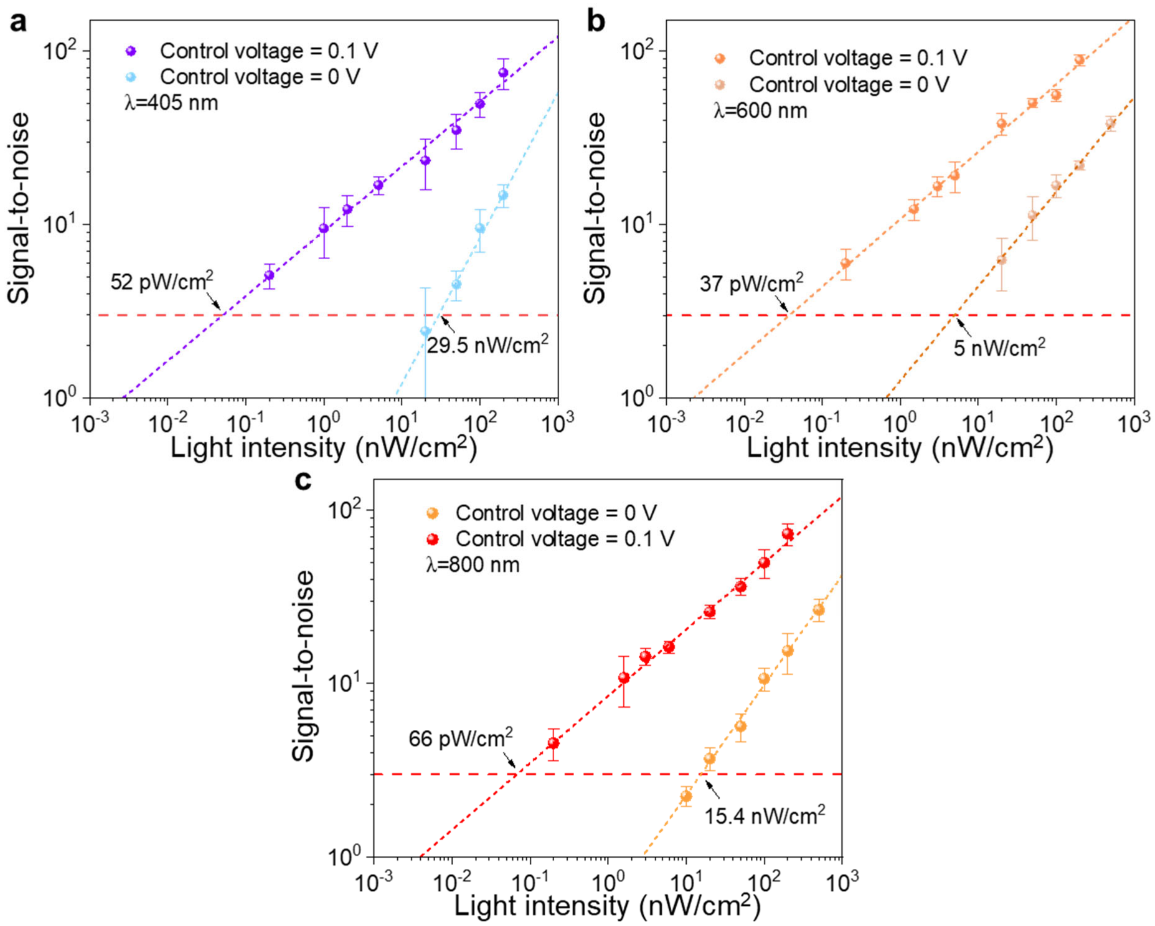

Supplementary Figure 14 | SNR of the FPD under different light intensities. The error bars represent standard deviation and are calculated according to variations in the current signal. The SNR of the FPD under the light wavelengths of 405 **(a)**, 600 **(b),** and 800 nm **(c)** illumination. The red dashed line represents an SNR of 3.

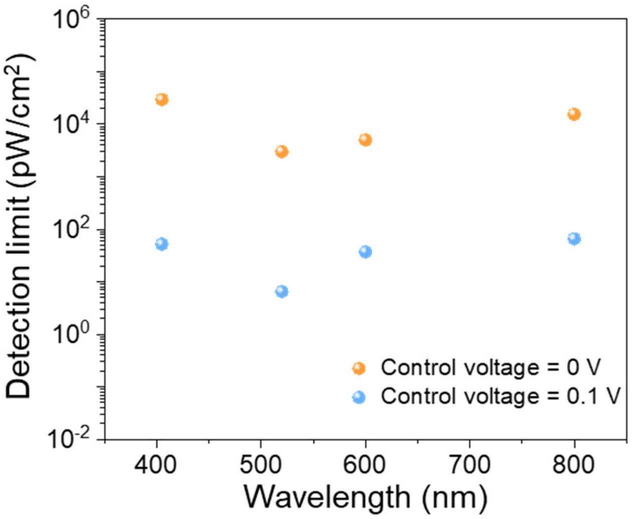

Supplementary Figure 15 | LoD of the FPD at different light wavelengths.

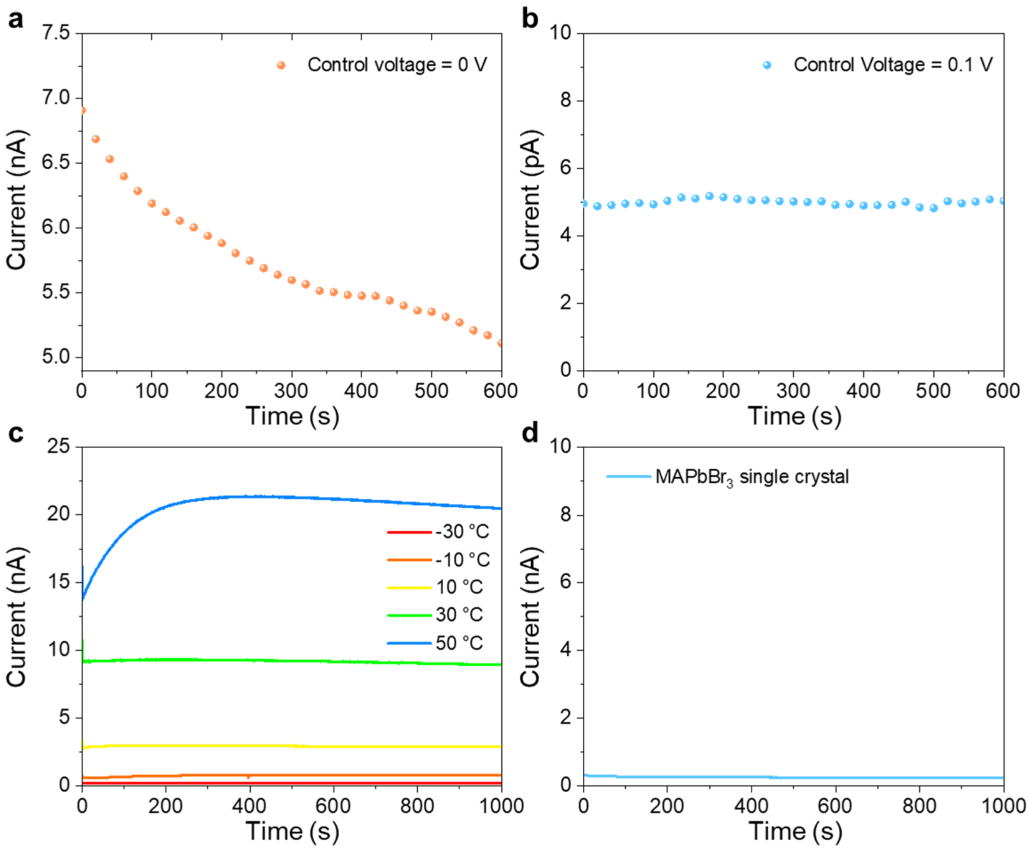

Supplementary Figure 16 | The origin of FPD's baseline drift. a,**b** The relationship between the baseline drift and time extracted from Fig. 3g,h. **c**, Current-time curves of baseline drift at different temperatures (Control voltage is disabled). **d**, Current-time curve of baseline drift of $MAPbBr_3$ single crystal perovskite.

Due to the weak ionic-bonding nature of perovskites, ions tend to migrate under electrical bias, which leads to ionic conductivity and further causes baseline drift[1]. By control voltage modulation, not only the value of the dark current is reduced, but also the ion migration near the signal electrode is suppressed, thus rendering a more stable baseline.

Furthermore, both crystal defects and high temperature are the origins for promoting ion migration and thus lead to baseline drift[2]. As shown in Supplementary Fig. 16c, as the test temperature increases, the dark current increases and the baseline drift becomes more obvious. The relationship between the ion migration rate (r_m) and temperature (T) can be expressed by the following expression:

$$r_m \propto \exp\left[-\frac{E_A}{k_B T}\right]$$

Where E_A is activation energy and k_B is Boltzmann's constant. In addition, ion migration is more likely to occur in polycrystalline films with many crystal defects due to the smaller ion activation energy at defect sites[3]. For comparison, we tested the baseline drift curve of $MAPbBr_3$ single crystal perovskite, and due to its lower defect density, the baseline current value of single crystal perovskite is very small (~0.3 nA) and there is basically no significant drift (Supplementary Fig. 16d). Therefore, both crystal defects and high temperature are the reasons for promoting ion migration and thus lead to baseline drift.

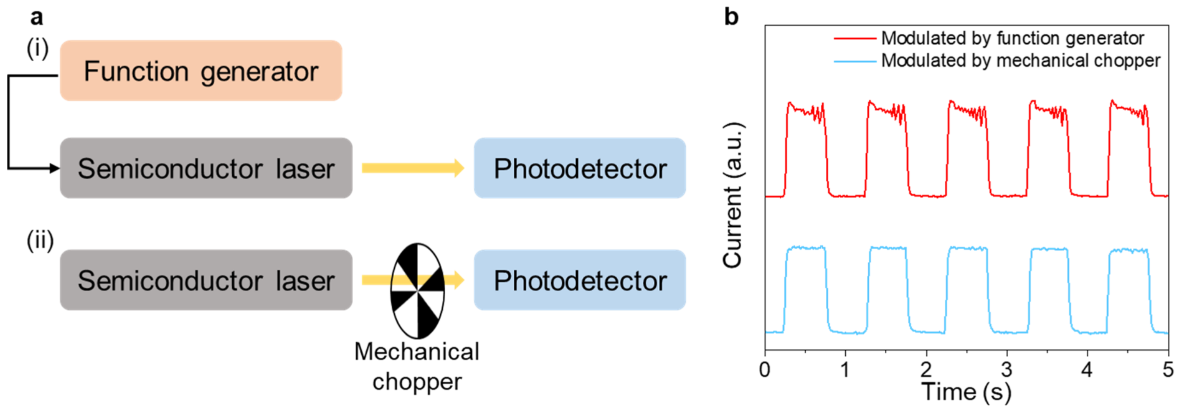

Supplementary Figure 17 | The photocurrent of FPD obtained by pulses light illumination with different modulation methods. a, Light pulse generation method and **b**, corresponding photocurrent signal.

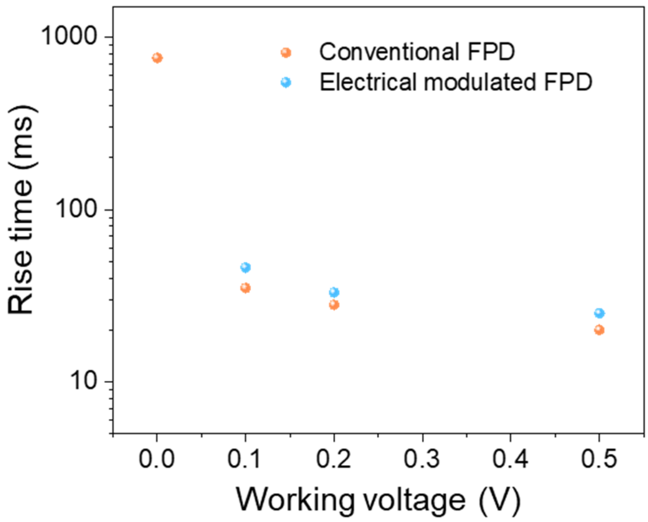

Supplementary Figure 18 | Relationship between the response time and working voltage of FPD with/without control voltage modulation.

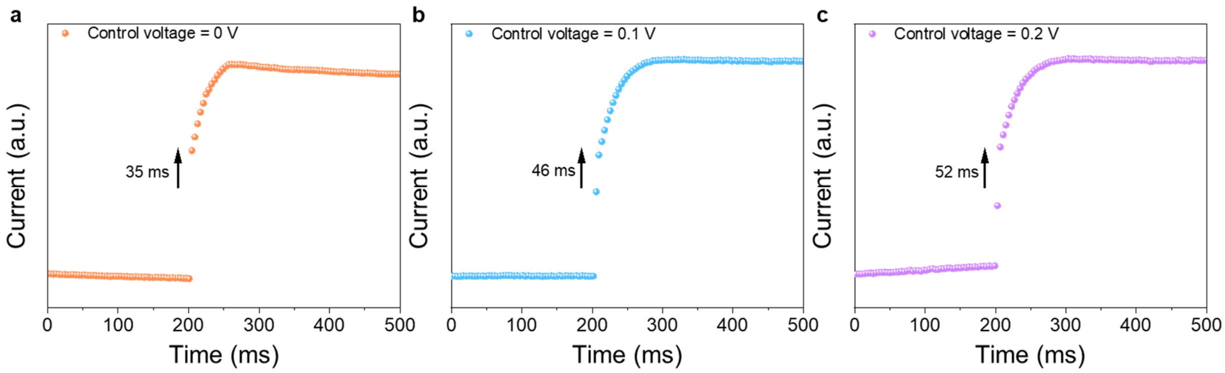

Supplementary Figure 19 | Response time of FPD under 0, 0.1, 0.2 V control voltage modulation, when the working voltage is fixed at 0.1 V.

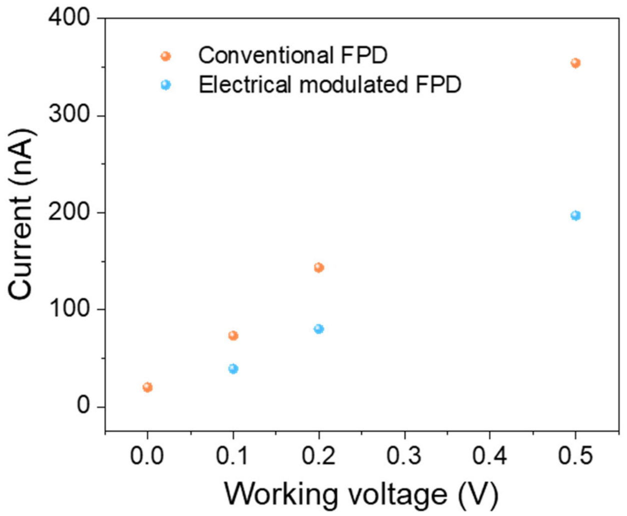

Supplementary Figure 20 | Photocurrent of conventional and electrical modulated FPD under different working voltage.

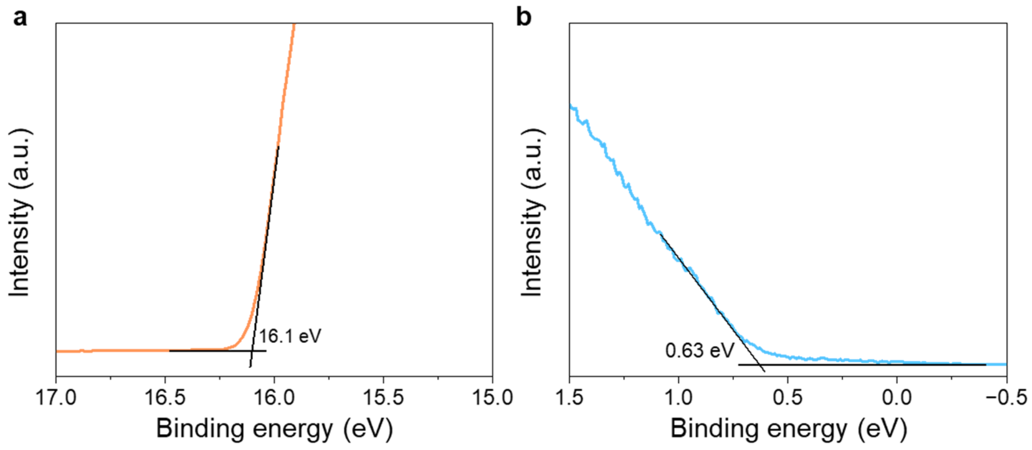

Supplementary Figure 21| Ultra-violet photoelectron spectroscopy (UPS) of perovskite. **a**, The secondary electron cutoff region **b**, and the valence band onset of UPS of perovskite film. The Fermi level, valance band maximum (VBM) and conduction band minimum (CBM) of perovskite were extracted at -5.12, -5.75, and -4.21 eV, respectively.

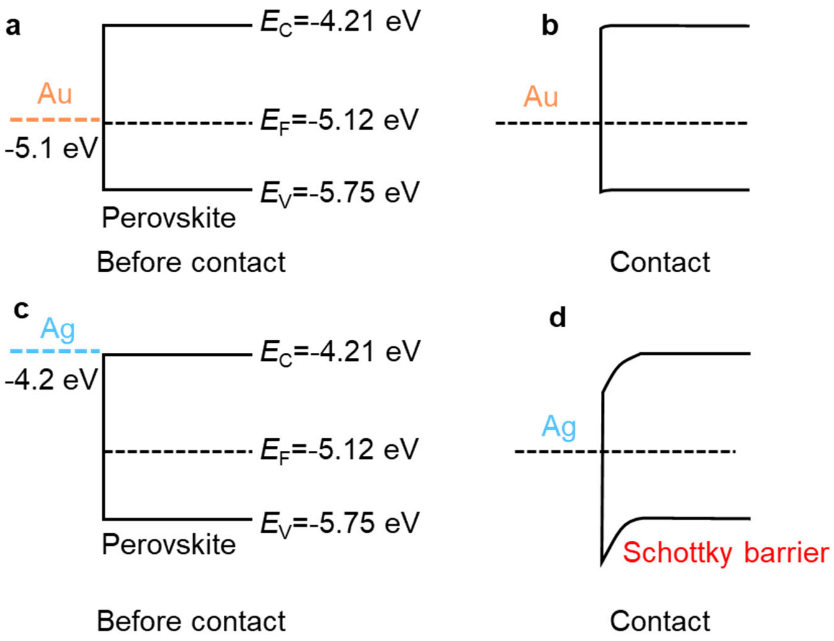

Supplementary Figure 22 | Energy band diagrams for the Au/perovskite and Ag/perovskite interface. When two metals with different work functions, Au (-5.1 eV) and Ag (-4.2 eV)[5], form contacts with perovskite, different injection barriers could be expected. As shown in the energy band diagram, perovskite materials forms Schottky contacts with Ag, but could form Ohmic contact with Au. There exists an obvious Schottky barrier between perovskite and Ag electrode, which hinders the transport of charge carrier[6]. However, since the work function of the Au electrode (-5.1 eV) is close to the Fermi level of the perovskite (-5.12 eV), there is almost no barrier at the Au/perovskite interface, which facilitates the carrier transport.

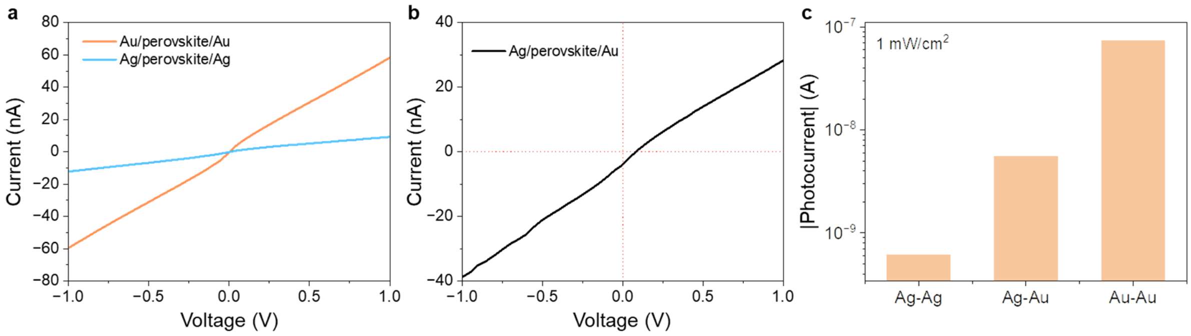

Supplementary Figure 23 | Characteristics of the FPD fabricated by different electrodes. **a**, Current-voltage characteristics of Au/perovskite/Au and Ag/perovskite/Ag FPDs. The contact resistance with Ag electrode is much larger than that of Au electrodes, which is caused by the high Schottky barrier at the Ag electrode/perovskite interface. **b**, Current-voltage characteristics of device with asymmetric electrodes of Ag/perovskite/Au. The current-voltage curve deviated from the origin, which also indicated the existence of the Schottky barrier. **c**, Photocurrent of FPD with different electrodes under 1 mW/cm^2 illumination. Device with both Au electrodes exhibited the highest photocurrent than symmetric Ag-Ag or asymmetric Ag-Au electrodes.

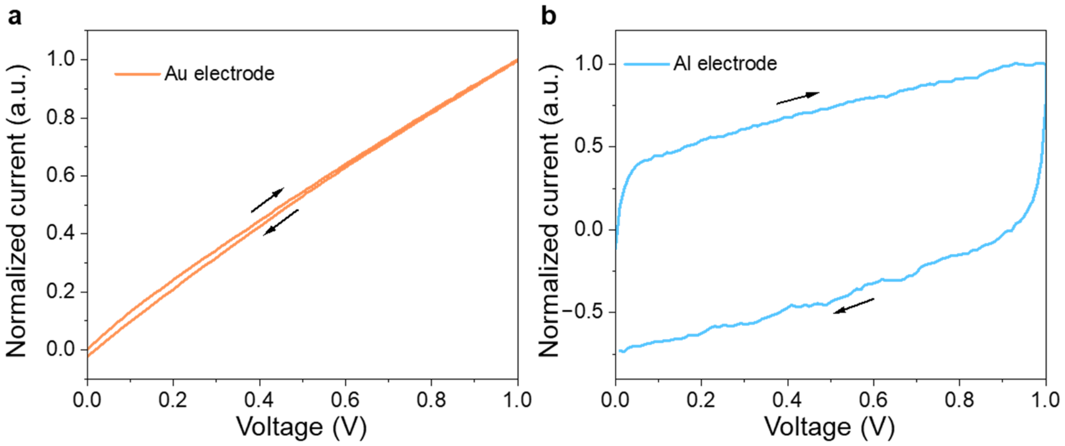

Supplementary Figure 24 | Forward and reverse current-voltage scan curves of FPD with Au and Al electrodes at the dark condition. Due to the smaller hysteresis and more stable chemical properties, Au electrodes were utilized to explore the effect of electric field modulation.

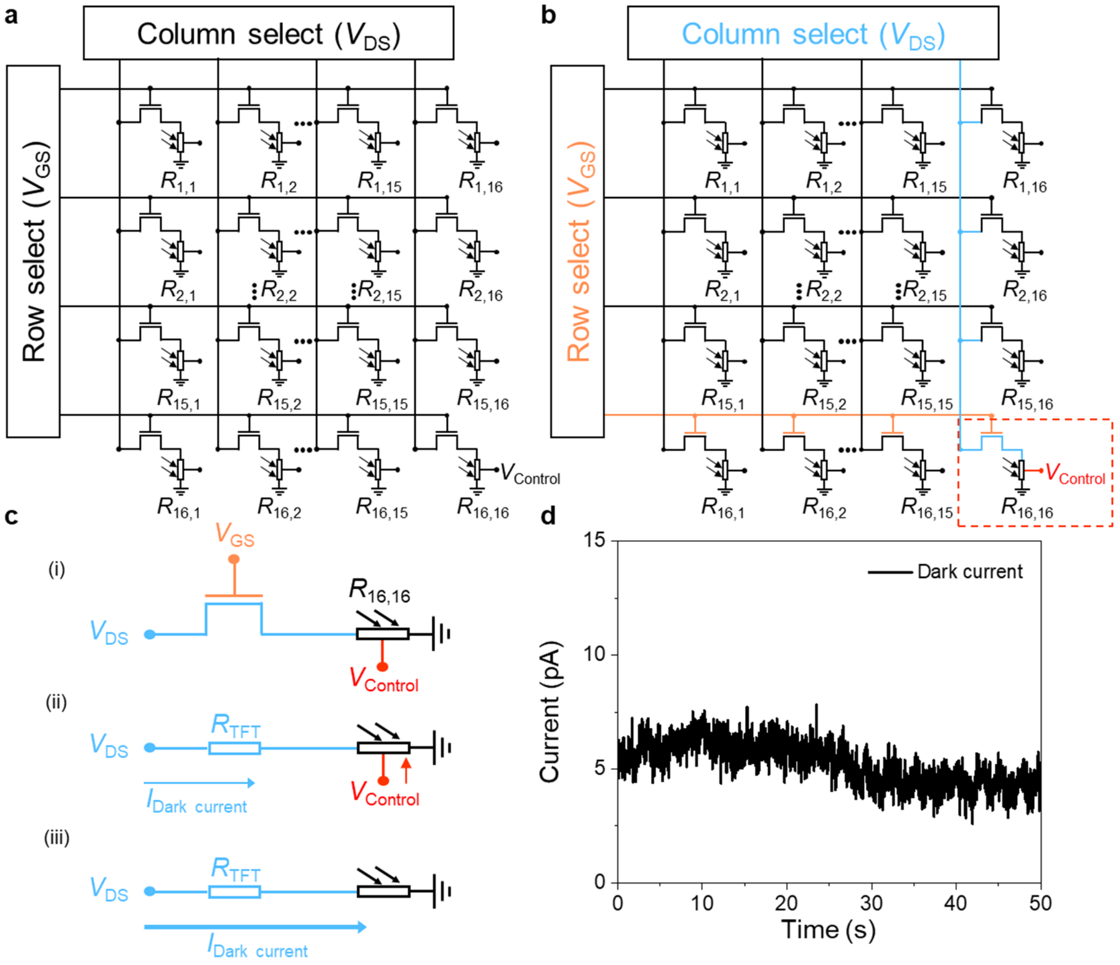

Supplementary Figure 25 | A detailed analysis of the dark current and noise of the array. a, Circuit diagram of the sensor array. **b**, Take $R_{16,16}$ as an example to analyze the addressing process and the origin of dark current. The gate voltage (V_{GS}=20 V) and drain voltage (V_{DS}=0.1 V) are respectively applied to the sixteenth row (orange line) and sixteenth column (blue line) to read the signal of $R_{16,16}$. **c**, A simplified circuit of the case of the addressed single pixel. **d**, Current-time curve of a single pixel measured in the array.

We use a transistor in series with a perovskite photodetector to construct an active matrix FPD (AM-FPD) array, and the corresponding equivalent circuit diagram is shown in Supplementary Fig. 25. During the testing process, the corresponding row and column electrodes are selected through matrix switch module (NI PXIe-2531) to turn on a specific transistor, thereby reading the perovskite photodetector signal. Take $R_{16,16}$ as an example to analyze the addressing process and the origin of dark current. The gate voltage (V_{GS}=20 V) and drain voltage (V_{DS}=0.1 V) are

respectively applied to the sixteenth row (orange line) and sixteenth column (blue line) to read the signal of $R_{16,16}$. Since the other transistors are in the off state (the gate voltage is not applied), the current in the circuit is almost all contributed by $R_{16,16}$ at this time. The simplified equivalent circuit diagram of the addressed single pixel is shown in Supplementary Fig. 25ci. The transistor is turned on at this time, so the transistor can be equivalent to a resistor (R_{TFT}), whose resistance value is much smaller than the perovskite photoconductor ($R_{TFT} \ll R_{16,16}$). As shown in Supplementary Fig. 25cii and 25ciii, when the control voltage is applied, the dark current (blue arrow, $I_{Dark\ current}$) measured by the instrument is shunted by the control electrode, so the measured $I_{Dark\ current}$ value can be significantly reduced.

In the actual test, the absolute value of the measured dark current can also be reduced to ~5 pA, but its random noise is significantly higher than the current signal measured directly on the perovskite, which is mainly due to the environmental noise and transistor device noise (Supplementary Fig. 25d). In future applications, the detection performance of the array can be improved by improving the quality of addressing transistors, optimizing the circuit wiring structure, and enhancing the electromagnetic shielding ability of the device to obtain higher-quality current data.

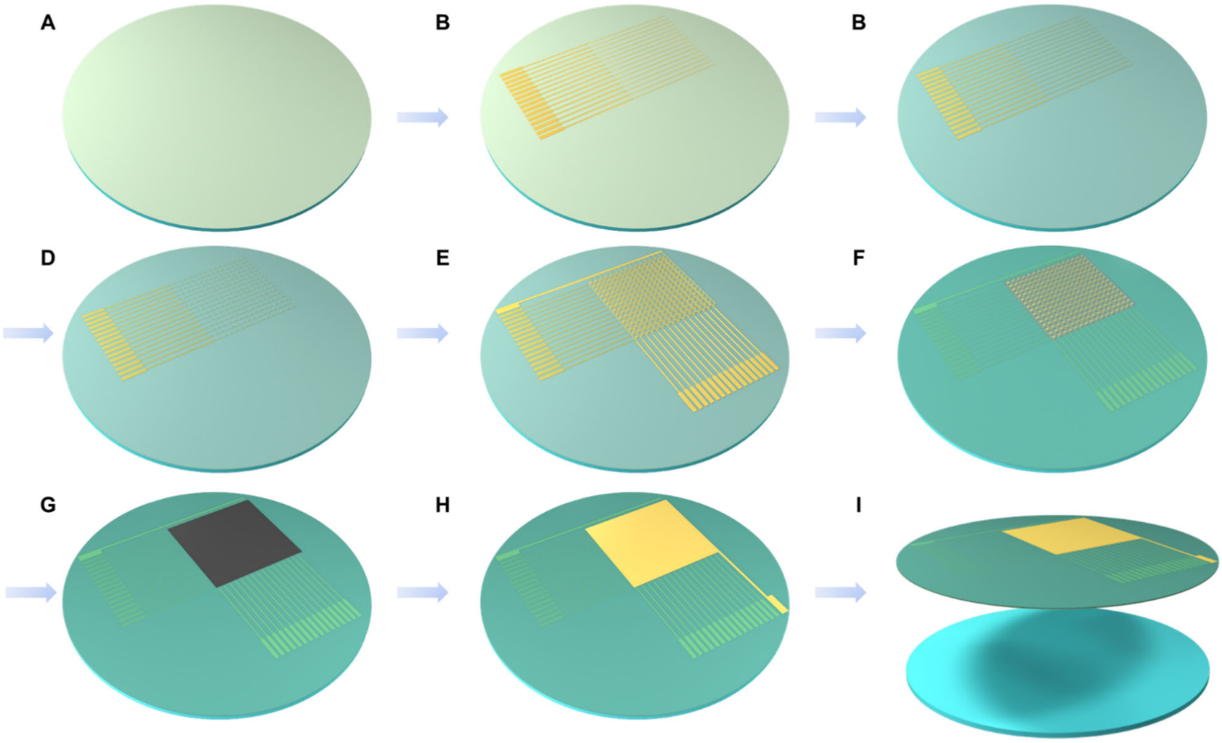

Supplementary Figure 26 | Fabrication process of the AM-FPD array. a, The CPI film deposited on Si/SiO_2 substrate. **b**, Deposition and patterning of gate electrodes (Ni/Au, 5/30 nm) **c**, Deposition of SiO_2 (100 nm) dielectric layer. **d**, Deposition, patterning and annealing (300°C, 1 h) of the In_2O_3 channel layer. **e**, Deposition and pattering of Ni/Au (5/50 nm) source and drain electrodes. **f**, Deposition and patterning of the SU-8 passivation layer and CYTOP layer (for patterning perovskite). **g**, Deposition and patterning of the perovskite for light-sensing. **h**, Deposition and patterning of the Ni/Au (5/100 nm) control electrode. **i**, Peel-off of the AM-FPD array from the silicon wafer by electrochemical delamination method[7].

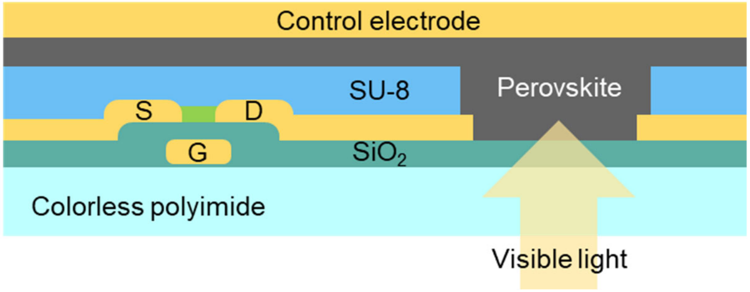

Supplementary Figure 27 | Cross-sectional of a single pixel of AM-PFD array.

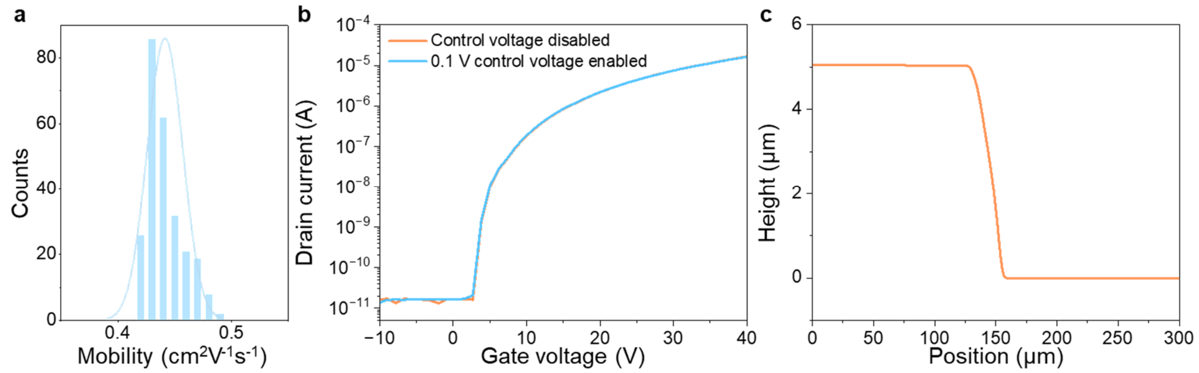

Supplementary Figure 28 | Effect of control voltage on transistor mobility. a, Statistical distributions of the mobility (average μ_{lin}=0.44 cm^2 V^{-1} s^{-1}) of 256 In_2O_3 TFTs. **b**, Transfer curves of the In_2O_3 TFT with and without a control voltage applied. **c**, The thickness of the SU-8 film was ~5 µm measured by a stylus profiler (P7, KLA-Tencor).

Supplementary Table 1. Summary of the reported flexible photoconductive-type perovskite photodetector arrays for image sensing (NS: nanosheet; MW: microwire; NW: nanowire).

Materials	Bias (V)	Electric field (V μm^{-1})	*D** (Jones)	*R* (A W^{-1})	Wavelength (nm)	Dark current (pA)	Sensor area (cm^2)	Pixel number	ref
$MAPbI_3$ MW network	10	0.05	1.02 ×10^{12}	0.1	650	70	2.5×2.5	7×7	8
single-crystalline aligned $MAPbI_3$ MW array	-5	-0.2	5.25 ×10^{12}	13.5	420	>1000	1×1	21×21	9
$MAPbI_3$ NW	0.3	-	≈1×10^{10}	≈0.035	450-780	-	1.28×1.28	32×32	10
$CsPbBr_3$ NSs	10	0.2	6.4×10^{8}	0.0449	450	-	72	1665	11
$MAPbI_{3-x}Cl_x$	5	-	9.4×10^{11}	2.17	650	1.14 ±0.64×10^{3}	≈0.5×0.5	10×10	12
$CsPbBr_3$	5	-	3.94×10^{12}	3.15	450	0.33± 0.19×10^{3}	≈0.5×0.5	10×10	13
$CsPbBr_3$	9	0.045	-	2.1	442	10000 (under 5 V)	-	8×4	14
$MAPbI_3$	5	-	7.1×10^{10}	0.0341	405	-	-	8×10	15
$MAPbX_3$	3	1.5	1.41×10^{12}	14.97	530	-	-	10×10	16
$MAPbI_{3-x}Cl_x$	3	-	2.45×10^{8}	1.24×10^{-5}	300-800	-	≈2.5×2.5	10×10	17
$FAMAPb(Br_{0.4}I_{0.6})_3$	3	0.04	3.25×10^{11}	0.0452	-	9.6 × 10^{4}	-	10×10	18
$CsPbBr_3$	10	0.1	3.5×10^{12}	1.1	500	20	-	5×5	19
$MAPbBr_3$ MW crystal	1	0.01	1.2×10^{14}	414	365	>20	-	5×4	20
Cs_2SnI_6	5	0.05	3.02×10^{10}	1.58	455	>10^{6}	2×2	3×3	21
$MAPbBr_3$	3	0.01	-	0.0042	405	-	-	6×6	22
**$FA_{0.92}Cs_{0.04}MA_{0.04}PbI_3$**	**0.1**	**0.001**	**1.12×10^{14}**	**8**	**520**	**~5**	**2×2**	**16×16**	**This work**

Supplementary References

1. Eames, C., Frost, J. M., Barnes, P. R., O'Regan, B. C., Walsh, A., Islam, M. S. Ionic transport in hybrid lead iodide perovskite solar cells. *Nat. Commun.* **6**, 7497 (2015).
2. Yuan, Y.*, et al.* Electric-field-driven reversible conversion between methylammonium lead triiodide perovskites and lead iodide at elevated temperatures. *Adv. Energy Mater.* **6**, 1501803 (2016).
3. Shao, Y.*, et al.* Grain boundary dominated ion migration in polycrystalline organic–inorganic halide perovskite films. *Energy Environ. Sci.* **9**, 1752-1759 (2016).
4. Kahn, A. Fermi level, work function and vacuum level. *Mater. Horiz.* **3**, 7-10 (2016).
5. Li, D.*, et al.* Metal halide perovskite/electrode contacts in charge-transporting-layer-free devices. *Adv. Sci.* **9**, 2203683 (2022).
6. Zhang, Z., Yates, J. T., Jr. Band bending in semiconductors: chemical and physical consequences at surfaces and interfaces. *Chem. Rev.* **112**, 5520-5551 (2012).
7. Zhang, H., Liu, Y., Yang, C., Xiang, L., Hu, Y., Peng, L. M. Wafer-scale fabrication of ultrathin flexible electronic systems via capillary-assisted electrochemical delamination. *Adv. Mater.* **30**, e1805408 (2018).
8. Deng, H.*, et al.* Flexible and semitransparent organolead triiodide perovskite network photodetector arrays with high stability. *Nano Lett.* **15**, 7963-7969 (2015).
9. Deng, W.*, et al.* Aligned single-crystalline perovskite microwire arrays for high-performance flexible image sensors with long-term stability. *Adv. Mater.* **28**, 2201-2208 (2016).
10. Gu, L.*, et al.* 3D arrays of 1024-pixel image sensors based on lead halide perovskite nanowires. *Adv. Mater.* **28**, 9713-9721 (2016).
11. Deng, W.*, et al.* All-sprayed-processable, large-area, and flexible perovskite/MXene-based photodetector arrays for photocommunication. *Adv. Opt. Mater.* **7**, 1801521 (2019).
12. Wu, W.*, et al.* Flexible photodetector arrays based on patterned $CH_3NH_3PbI_{3-x}Cl_x$ perovskite film for real-time photosensing and imaging. *Adv. Mater.* **31**, e1805913 (2019).
13. Wu, W.*, et al.* Ultrathin and conformable lead halide perovskite photodetector arrays for potential application in retina-like vision sensing. *Adv. Mater.* **33**, e2006006 (2021).
14. Cao, F.*, et al.* Highly stable and flexible photodetector arrays based on low dimensional $CsPbBr_3$ microcrystals and on-paper pencil-drawn electrodes. *J. Mater. Chem. C* **5**, 7441-7445 (2017).
15. Xia, B.*, et al.* Flexible metal halide perovskite photodetector arrays via photolithography and dry lift-off patterning. *Adv. Eng. Mater.* **24**, 2100930 (2021).
16. Wang, Q., Zhang, G., Zhang, H., Duan, Y., Yin, Z., Huang, Y. High-resolution, flexible, and full-color perovskite image photodetector via electrohydrodynamic printing of ionic-liquid-based ink. *Adv. Funct. Mater.* **31**, 2100857 (2021).
17. Jang, J.*, et al.* 3D heterogeneous device arrays for multiplexed sensing platforms using transfer of perovskites. *Adv. Mater.* **33**, 2101093 (2021).

18. Kim, T., Jeong, S., Kim, K. H., Shim, H., Kim, D., Kim, H. J. Engineered surface halide defects by two-dimensional perovskite passivation for deformable intelligent photodetectors. *ACS Appl. Mater. Interfaces* **14**, 26004-26013 (2022).

19. Chen, Y., Zhao, C., Zhang, T., Wu, X., Zhang, W., Ding, S. J. Flexible and filter‐free color‐imaging sensors with multicomponent perovskites deposited using enhanced vapor technology. *Small* **17**, 2007543 (2021).

20. Li, S. X., Xia, H., Sun, X. C., An, Y., Zhu, H., Sun, H. B. Curved photodetectors based on perovskite microwire arrays via in situ conformal nanoimprinting. *Adv. Funct. Mater.* **32**, 2202277 (2022).

21. Krishnaiah, M., Kim, S., Kumar, A., Mishra, D., Seo, S. G., Jin, S. H. Physically detachable and operationally stable Cs_2SnI_6 photodetector arrays integrated with μ-LEDs for broadband flexible optical systems. *Adv. Mater.* **34**, e2109673 (2022).

22. Zhang, T., *et al.* Pen-writing high-quality perovskite films and degradable optoelectronic devices. *RSC Adv.* **12**, 3924-3930 (2022).
